# Metabolic engineering of *Escherichia coli* BL21 strain using simplified CRISPR-Cas9 and asymmetric homology arms recombineering

**DOI:** 10.1186/s12934-022-01746-z

**Published:** 2022-02-05

**Authors:** Sudha Shukal, Xiao Hui Lim, Congqiang Zhang, Xixian Chen

**Affiliations:** Singapore Institute of Food and Biotechnology Innovation (SIFBI), Agency for Science, Technology and Research (A*STAR), Proteos level 4, Singapore, 138673 Singapore

**Keywords:** CRISPR-Cas9, Asymmetric homology arms, Lycopene, Cell size, Triacylglycerol pathway, Acetyl-CoA availability

## Abstract

**Background:**

The recent CRISPR-Cas coupled with λ recombinase mediated genome recombineering has become a common laboratory practice to modify bacterial genomes. It requires supplying a template DNA with homology arms for precise genome editing. However, generation of homology arms is a time-consuming, costly and inefficient process that is often overlooked.

**Results:**

In this study, we first optimized a CRISPR-Cas genome engineering protocol in the *Escherichia coli (E. coli)* BL21 strain and successfully deleted 10 kb of DNA from the genome in one round of editing. To further simplify the protocol, asymmetric homology arms were produced by PCR in a single step with two primers and then purified using a desalting column. Unlike conventional homology arms that are prepared through overlapping PCR, cloning into a plasmid or annealing synthetic DNA fragments, our method significantly both shortened the time taken and reduced the cost of homology arm preparation. To test the robustness of the optimized workflow, we successfully deleted 26 / 27 genes across the BL21 genome. Noteworthy, gRNA design is important for the CRISPR-Cas system and a general heuristic gRNA design has been proposed in this study. To apply our established protocol, we targeted 16 genes and iteratively deleted 7 genes from BL21 genome. The resulting strain increased lycopene yield by ~ threefold.

**Conclusions:**

Our work has optimized the homology arms design for gene deletion in BL21. The protocol efficiently edited BL21 to improve lycopene production. The same workflow is applicable to any *E. coli* strain in which genome engineering would be useful to further increase metabolite production.

**Supplementary Information:**

The online version contains supplementary material available at 10.1186/s12934-022-01746-z.

## Introduction

*Escherichia coli* BL21 strain is one of the most utilized bacterial platforms for recombinant protein production and metabolic engineering [1–3]. The ability to easily and efficiently modify the genome of BL21 is highly desirable to further improve strain performance for industrial applications. With the breakthrough in genome editing technology, clustered regularly interspaced short palindromic repeats (CRISPR) and associated proteins (Cas) system have become a common genome editing tool applied from microbes to mammals [4]. CRISPR-Cas9, a class 2 type II system, is well-characterized and the most widely applied because of its simple design [1]. It consists of a single Cas9 nuclease protein complex with a CRISPR RNA (crRNA) and trans-acting crRNA (tracrRNA) duplex or a single guide RNA (sgRNA) with fused 3’ end of crRNA and 5’ end of tracrRNA [5]. The complex specifically targets the protospacer DNA which is complementary to crRNA and generates a double-strand (ds) break via the recognition of a protospacer adjacent motif (PAM) sequence, 5’-NGG-3’ [5]. Subsequently, the dsDNA break (DSB) can be repaired via homologous recombination (HR) or non-homologous end-joining (NHEJ). A donor DNA is required for specific deletion, insertion, or mutation of the genome sequence during HR. In *E.coli*, λ-Red recombinases are often used for efficient recombination of the chromosome and donor DNA [6, 7].

**Fig. 1:**
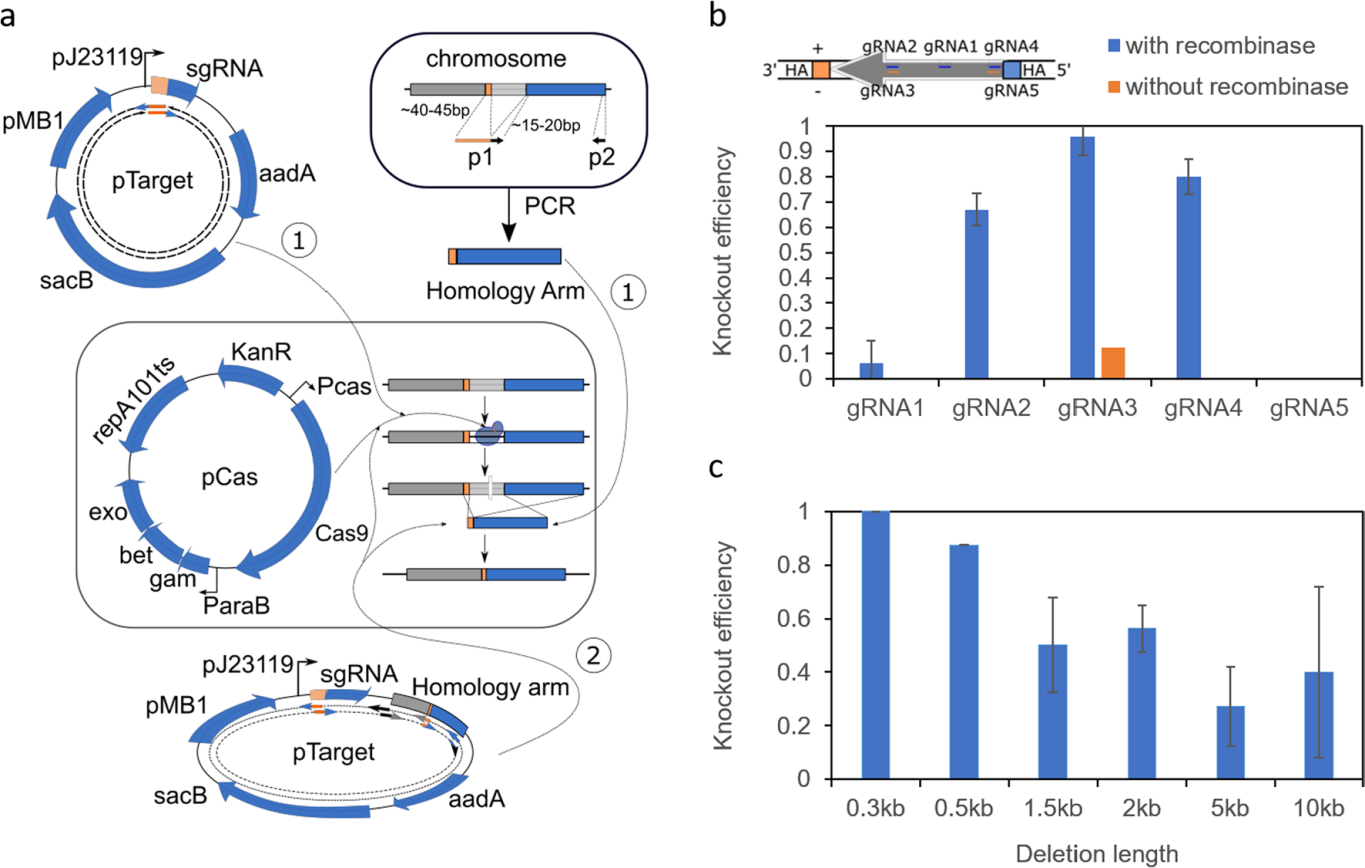
The modified CRISPR-Cas9 based genome deletion in the *E. coli* BL21 strain. **a** Schematic representation of the modified two-plasmid system adapted from Jiang et al. [6]. Two different methods have been tested. Both requires the pCas plasmid being transformed into *E. coli* cell first. Subsequently, the pTarget plasmid with homology arm either as PCR fragments (method 1) or carried on pTarget plasmid (method 2) were transformed into the cell. For method 1, the homology arm is obtained with a simple PCR step where the forward primer 1 (p1) carries the upstream (40–45 bp) homologous sequence fused with the downstream (15-20 bp) homologous sequence for priming, and the reverse primer 2 (p2) is about 15–20 bp targeting 500 bp downstream of p1 priming sequence. The total length of p1 primer is 60 bp to ensure efficient synthesis and PCR. The gRNA sequence is changed with restriction-free cloning method [23]. In total, 4 primers were used for each target gene modification. For method 2, to clone the pTarget plasmid, 4 PCR fragments with 8 primers are used to assemble the plasmid. **b** Knockout efficiency for CRISPR-Cas9 method 2 in BL21 cell targeting the *adhE* gene. Five different gRNA designs were tested where their targeting positions are illustrated. Knockout efficiency is calculated based on the number of colonies with successful deletion over the total number of colonies tested. **c** Knockout efficiency for deleting various lengths from the BL21 genome using gRNA3 targeting *adhE* region. All the efficiencies were obtained through replicate experiments

Many pioneering works in bacteria optimized the CRISPR-Cas system in the *E. coli* K-12 strain [6, 8, 9]. For example, the widely used two-plasmid system (pTarget and pCas) developed by Jiang and co-workers utilizes CRISPR-Cas9 and λ-Red recombination for scarless genome modification in *E. coli* K-12 strain MG1655 [6]. While this manuscript was in preparation, the same group published a modified pCas/pTarget system as the original two-plasmid system failed to work in BL21 [10]. Indeed, very few reports have shown an optimized CRISPR-cas system in BL21, except a large-scale validation study by Zerbini and co-workers which has shown CRISPR-Cas mediated gene knockout in the BL21 Δ*ompA* strain [11]. In that study, 1–10 μg of synthetic DNA was used as homology arms for recombineering, which is costly and restricts the homology arm length to be 70–120 bp. In the two-plasmid system, the Cas9 and λ-Red recombinases are co-expressed on pCas plasmid, whereas the donor DNA and sgRNA are carried on the pTarget plasmid (Fig. 1a). Cloning of the pTarget plasmid would take at least 3 days to assemble four DNA fragments. The authors noted that cloning pTarget plasmid became complicated when multiple donor DNAs were included. They attempted to simplify the procedure by using PCR fragments as donor DNAs. However, the recombination efficiency was significantly decreased when the homology arm length was shortened from 400 to 40 bp [6].

In this study, we have improved the CRISPR-Cas mediated deletion in BL21 and optimized the homology arms design. Instead of symmetrical homology arms, we applied asymmetric homology arms, which can be obtained in a single PCR step. In addition, only 100 ng of donor DNA was transformed to edit the genome. We have validated the optimized protocol with 27 gene targets across the BL21 genome and achieved successful gene deletion up to 3.4 kb. 18 out of 27 genes achieved ≥ 75% knockout efficiencies. We termed the workflow as CRASH (*CR*ISPR-cas9 and *as*ymmetric *h*omology arm mediated genome modification). Lastly, we applied the CRASH protocol to iteratively modify the BL21 genome. By increasing cell size, regulating triacylglycerol (TAG) production, and redirecting acetyl-CoA flux to the mevalonate pathway, we have successfully improved lycopene yield by ~ threefold.

## Results

### Optimization of CRISPR-Cas9 knock-out in E. coli B strain

We adapted the two-plasmid system developed by Jiang and co-workers to BL21 [6, 11], in which Cas9 and sgRNA were overexpressed on the pCas and pTarget plasmid, respectively (Fig. 1a). Curing of pTarget plasmid was modified to overexpress the toxic *sacB* gene from *Bacillus subtilis* in the presence of 5% sucrose (Fig. 1a) [11]. The gRNA-pMB1 expression cassette from pCas plasmid was removed. To establish the protocol, five gRNAs targeting *adhE* were tested first, based on the distance from the PAM sequence to the nearer homology arm sequence (PAM-to-HA distance). Four gRNAs (gRNA2, gRNA3, gRNA4 and gRNA5) were within 30 bp for the PAM-to-HA distance, while gRNA1 was > 130 bp away from the nearer homology arm sequence (Table 1). Moreover, gRNA2-5 were targeting different DNA strands at the 5’ and 3’ regions of the *adhE* gene (Fig. 1b). All of the five gRNAs were cloned into the pTarget plasmid which carried the 500 bp homology arm sequences (Fig. 1a, method 2). The five pTarget plasmids were then transformed into *E. coli* BL21 cells overexpressing *cas9* and λ-recombinase genes and plated on agar plates with kanamycin and spectinomycin antibiotics. Both the gRNA targeting sequence and PAM sequence were deleted after homologous recombination, and the colonies formed on agar plate were expected to have genome modifications. After overnight incubation, 8 colonies from each agar plate were picked and analysed by colony-PCR. As shown in Fig. 1b, only gRNA3 was effective to achieve nearly 100% knockout efficiency. The knockout efficiencies were ranked to be gRNA3 > gRNA4 > gRNA2 > gRNA1 > gRNA5. Hardly any successful gene deletion was observed when either gRNA1 or gRNA5 was used. Although fewer colonies were formed on gRNA1 and gRNA5 agar plates (Additional file 1: Table S1), there were many “escapers” where the targeted genome region remained intact [12]. We hypothesized that the activities of the recombinases were insufficient, and the endogenous recA-mediated SOS response rescued cells from CRISPR-cas9 induced cell death [13]. However, overexpressing recA56, the dominant negative form of recA, did not improve the knockout efficiency when gRNA1 was used (Additional file 1: Table S2, pCas-V6). Replacing arabinose promoter with a stronger T7 promoter to overexpress λ-recombinase genes did not yield successful knock out clones using gRNA1. In fact, it reduced the colony forming units or transformation efficiencies significantly for both gRNA1 and gRNA3 (Additional file 1: Table S2, pCas-V2). Moreover, when λ-recombinase was only expressed under leaky T7 expression, similar knockout efficiency was observed for gRNA3 as compared to induced λ-recombinase activity (Additional file 1: Table S2, pCas-V2). Similarly, when λ-recombinase genes were removed from pCas plasmids, one out of 8 colonies was successfully deleted when gRNA3 was introduced into BL21 cells overexpressing Cas9 only (Fig. 1b). These observations reiterated the importance of gRNA designs which may be the critical bottleneck in the CRISPR-Cas9 system. It is worth noting that in silico gRNA design tools predicted gRNA3 being the lowest efficiency among the 5 gRNAs tested (Additional file 1: Table S1) [14], indicating that there were unidentified factors governing the efficiencies of gRNAs.

**Table 1:** Target genes and gRNA designs used in the study with reference to Fig. 3

Gene	Gene direction	gRNA targeting Strand	Proximate gene end	gRNA sequence	Deletion size	PAM genomic Position	PAM-to-HA distance	knockout efficiency
adhE	Reverse	+	3'	ccttccctgactctgggttg	300	1283650	138	1/15
	Reverse	+	3'	aaatctatctacttccgccg	300	1283521	9	10/16
	Reverse	−	3'	cggcggaagtagatagattt	300	1283542	29	14/15
	Reverse	+	5'	ctcttgcctgtacactgacc	300	1283789	23	12/16
	Reverse	−	5'	ggttatcctggtcagtgtac	300	1283801	12	0/16
aroA	Forward	−	3'	gcagctggcgcggattagcc	1284	965175	11	2/9
	Forward	+	3'	gccagctgctcgaaataatc	1284	965143	43	5/10
	Forward	+	5'	agcgatgggttgtaacgtca	1284	963911	10	3/4
	Forward	−	5'	gttgtagagagttgagttca	1284	963903	1	0/4
zapB	Forward	−	5'	attagaagtgtttgagaaac	246	4026469	31	4/4
	Forward	−	3'	gcaggccctgctgggtcgca	246	4026670	14	1/4
tnaA	Forward	+	5'	cacgaatgcggaacggttca	1416	3777548	23	4/8
	Forward	+	3'	ttaaacttctttcagttttg	1416	3778922	20	1/8
aroB	Reverse	−	5'	aaccagatgcgatggtaatt	1089	3378824	41	1/4
	Reverse	−	3'	ttgttacgctgattgacaat	1089	3377793	14	2/4
aroC	Reverse	−	3'	ttattcattttttaccagcg	1086	2333650	9	1/12
	Reverse	−	5'	cccgtgcgattcgccgaaag	1086	2334687	40	1/12
sdhABCD	Forward	+	5'	gtctgtaggtccagattaac	2497	713776	30	0/4
	Forward	+	3'	gcaacaacatcgacttgata	2497	716941	22	4/4
serA	Reverse	−	3'	aggggaattagtacagcaga	1233	2888049	13	2/4
	Reverse	−	5'	gaaggctttccagcgccttt	1233	2889207	7	0/4
serB	Forward	−	5'	ccttaatgcctaacattacc	969	4542265	15	3/4
	Forward	−	3'	ggggtattctgcatcctctc	969	4543197	22	3/4
adeD	Forward	+	5'	ggctaatgtgatgaaattta	1767	3732350	20	0/4
	Forward	+	3'	cgtgacttccagcgtagtga	1767	3734071	26	3/4
speD	Reverse	−	3'	aaataaatctggcggagcct	873	137573	20	3/4
	Reverse	−	5'	caatttcttatcttctcctt	873	138404	6	3/4
metJ	Reverse	+	5'	gtctcaatttattgacgaag	318	4036957	24	2/4
	Reverse	+	3'	ggggattaacccggagacgt	318	4036635	11	0/4
tatA	Forward	−	5'	ttaatcatcatctaccacag	317	3928846	37	3/4
	Forward	−	3'	ggcggatacgaatcaggaac	317	3929079	44	4/4
hold	Forward	+	3'	gcaaatttgttgccataacg	383	4526535	24	2/12
	Forward	+	5'	ctgtaactgccagtctcgtc	383	4526187	10	1/12
rodZ	Reverse	+	3'	cagtacagatccagtatcaa	943	2506397	27	0/4
	Reverse	+	5'	caaaatgaagcacttactac	943	2507280	33	0/4
envC	Forward	−	5'	aggcgattaataccatgaca	1282	3656045	29	3/4
	Forward	−	3'	gtcaatccacagccgtggtt	1282	3657289	10	2/4
pdxH	Reverse	−	3'	tccacgcatcattttcacgc	657	1662590	46	1/4
	Reverse	−	5'	acccgcctttggtgtattca	657	1663160	44	1/4
ybaS	Forward	−	5'	aaacaaattacagcaggcag	933	480819	31	4/4
	Forward	−	3'	gcatcggtcgctaagcaact	933	481696	18	4/4
gadC	Reverse	−	3'	tcattcatcacaatatagtg	1605	1524468	33	4/4
	Reverse	−	5'	ccctaaaacggtattcctgt	1605	1525998	39	4/4
dgkA	Forward	+	5'	ggtgaatccagtggtattat	369	4164015	4	7/8
	Forward	+	3'	cgaccataacaggatgcacc	369	4164345	32	4/8
Pta	Forward	+	5'	agggatcagcataataatac	2145	2302006	4	0/10
	Forward	+	3'	ctgaatcgcagtcagcgcga	2145	2304106	24	1/10
fadE	Reverse	−	5'	gattttgagtattctcgcta	1542	247852	5	3/4
	Reverse	−	3'	gattgccatcaccgttgaag	1542	246385	25	4/4
pflB	Reverse	+	3'	gctgactaaagaacagcagc	2283	956400	35	1/4
	Reverse	+	5'	atgaaaagttagccacagcc	2283	958612	33	3/4
poxB	Reverse	+	3'	tggcgaaaacgaactggcta	1719	914040	3	4/4
	Reverse	+	5'	tatcgccaaaacactcgaat	1719	915710	43	4/4
IdhA	Reverse	−	3'	ttaaaccagttcgttcgggc	990	1417433	0	4/4
	Reverse	−	5'	gtactgttttgtgctataaa	990	1418390	10	4/4
aceAB	Forward	+	5'	ccttgtgaaagccagttcat	2436	4123387	22	1/4
	Forward	+	3'	agtacggaagaagccttcac	2436	4125768	30	0/3
ackA-pta	Forward	+	5'	accatttactgcatcgatga	3422	2300780	55	2/2
	Forward	+	3'	ctgaatcgcagtcagcgcga	3422	2304106	38	1/1

Next, we challenged the CRISPR-cas9 system to knockout longer lengths of DNA from the BL21 genome. gRNA3 was used, the PAM-to-HA distance was kept at 29 bp, and only the homology arm sequence proximate to the 5’ or downstream of *adhE* was changed (Fig. 1b). Despite a decrease in knockout efficiency observed when the deletion length increased from 300 bp to 10 kb, > 15% knockout efficiency was achieved for 10 kb deletion (Fig. 1c). A longer length (15 kb) was not tested, due to the presence of an essential gene [15]. Deleting 10 kb DNA simultaneously knocked out ~ 10 chromosome genes from *E. coli* BL21 genome, which would be sufficient for subsequence genome modification.

### Optimization of asymmetric donor DNA

**Fig. 2:**
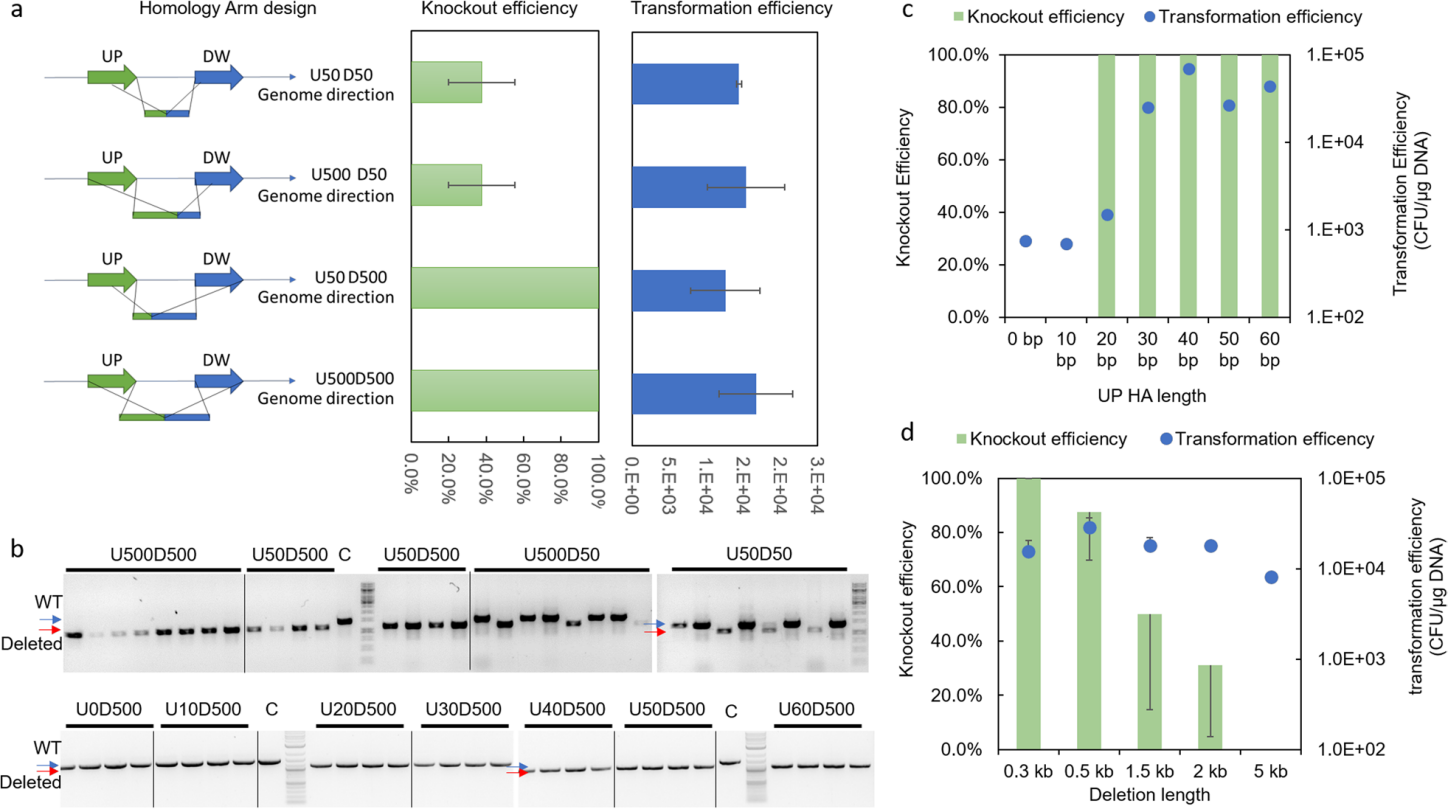
Optimizing the CRASH protocol targeting *adhE* gene with gRNA3. **a** Recombination efficiencies with four different homology arm (HA) designs. The U50D50 HA design carries 50 bp upstream homologous sequence and 50 bp downstream homologous sequence. The U500D50 HA design carries 500 bp upstream homologous sequence and 50 bp downstream homologous sequence. The U50D500 HA design carries 50 bp upstream homologous sequence and 500 bp downstream homologous sequence. The U500D500 HA design carries 500 bp upstream homologous sequence and 500 bp downstream homologous sequence. All are obtained via PCR using the pTarget-*adhE* plasmid carrying the homology arm as template. The template is completely removed before transforming the PCR products into BL21 cells for gene deletion. The number of colonies formed on the plates were counted by Qpix (Molecular Device) to determine the transformation efficiency. **b** Gel image of colony PCR results to check successfully deleted colonies. C is the PCR products obtained from non-edited cell. The size of non-edited (WT) and edited (Deleted) is indicated on the side of the gel. **c** Knockout efficiency and transformation efficiency with varied upstream HA length. The downstream HA length is kept at 500 bp. **d** Knockout efficiency and transformation efficiency for various lengths of DNA deleted from the BL21 genome. All the efficiencies were obtained with replicate experiments

While establishing the CRISPR-cas9 protocol, we realized that generating the pTarget plasmid with homology arms was a time-consuming step; at least 3 days were required to clone the pTarget plasmid [10, 16]. The cloning efficiency was lower, since four DNA fragments were amplified and assembled simultaneously (Fig. 1a, method 2). Reports have shown that double stranded DNA (dsDNA) could be used as an alternative donor DNA, and was more efficient than single stranded DNA (ssDNA) [6, 9]. Zerbini and co-workers validated a CRISPR-Cas9 protocol in BL21 ∆*ompA* strain, which required 1–10 μg of donor dsDNA to achieve genome modification [11]. dsDNA was generated by annealing two synthetic ssDNA, which inevitably increased the cost for donor DNA synthesis and limited donor DNA length. Here, we explored the use of asymmetric homology arms (aHA) as donor DNAs which can be obtained by one-step PCR (Fig. 1a). The efficiencies of aHA were tested with gRNA3 targeting *adhE* [17]. Four different designs of homology arm were tested: 1), 50 bp upstream of deletion site and 50 bp downstream of the deletion site (U50D50); 2), 500 bp upstream of deletion site and 50 bp downstream of the deletion site (U500D50); 3), 50 bp upstream of deletion site and 500 bp downstream of the deletion site (U50D500); 4), 500 bp upstream of deletion site and 500 bp downstream of the deletion site (U500D500) (Fig. 2a). ~ 100 ng of HA PCR products and pTarget-gRNA3 were transformed into BL21 cells overexpressing Cas9. All four HA designs resulted in similar transformation efficiencies (Fig. 2a). However only U50D500 and U500D500 HA gave rise to ~ 100% knockout efficiencies, whereas the other two HA designs only achieved ~ 40% knockout efficiencies (Fig. 2a, b). Richard et al. showed that the Cas9 protein asymmetrically released the PAM-distal nontarget strand after the double strand DNA break [17]. This may be the reason attributed to the preferred asymmetric design of HA. In addition, the same study demonstrated that 36 bp distal to the PAM was sufficient to achieve 60% knockout efficiency. Based on this, we systematically tested different lengths of HA upstream (UP) of the deletion site (0, 10, 20, 30, 40, 50 or 60 bp), while keeping the length of HA downstream (DW) of the deletion site to be 500 bp. As shown in Fig. 2c, transformation efficiencies decreased for UP HA length of 0, 10 and 20 bp. However, knockout efficiencies were maintained when the UP HA was ≥ 20 bp; the target 300 bp was successfully deleted in all four randomly picked colonies (Fig. 2b). As a result, shorter primers can be used to generate the aHA without compromising knockout efficiencies. We termed the method CRASH (*CR*ISPR-cas9 and *as*ymmetric *h*omology arm directed genomic engineering). With these encouraging results, we again challenged the CRASH protocol with aHA (U50D500) to knockout longer length of DNA from the BL21 genome. Both the knockout and transformation efficiencies decreased when the deletion length increased to 5 kb. Acceptable recombination efficiency, approximately 30%, was achieved when targeting to delete 2 kb. The length would be long enough to delete 1–2 genes from BL21 genome in one-step.

### gRNA design testing for multiple gene knockout targets

**Fig. 3:**
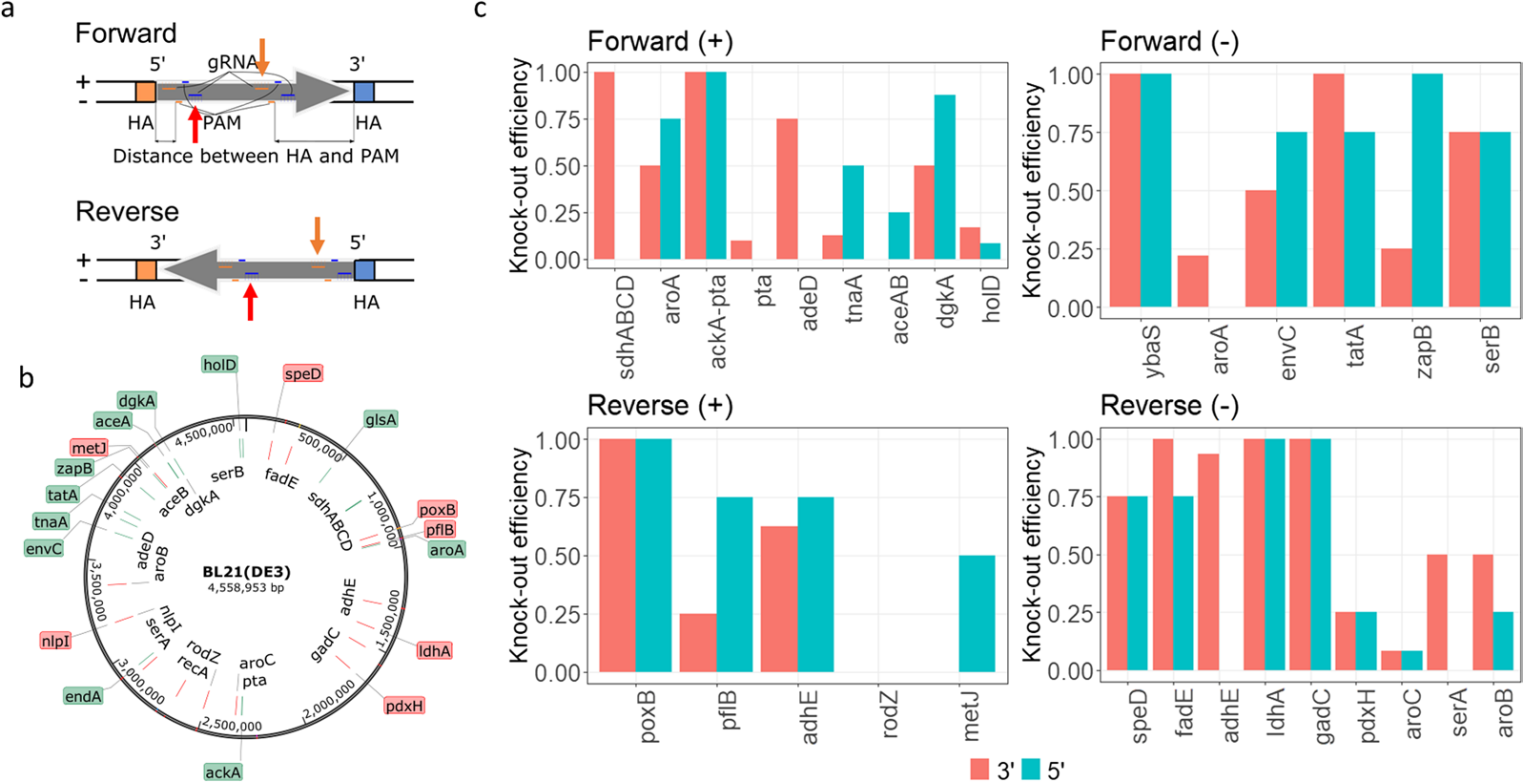
Testing gRNA designs and CRASH protocol across BL21 genome. **a** Illustrations of various gRNA designs. The red arrow indicates the most preferred gRNA design targeting the negative (−) strand of DNA. The orange arrow indicates the most preferred gRNA design targeting the positive ( +) strand of DNA. **b** Positions of the targeted genes along the BL21 genome. The reverse genes are labeled in red or anti-clockwise, and the forward genes are labeled in green or clockwise. **c** The knockout efficiencies for the various genes tested. Refer to Table 1 for details

To validate the CRASH protocol, we set to investigate gene deletion efficiencies across various positions along the BL21 genome (Fig. 3b). It is well known that gRNA design plays a key role in ensuring the success of CRISPR-cas9 mediated genome modifications [18, 19]. In silico design for gRNAs displays discrepancies with experimental results to some extent. Thus, we also examined at least two gRNA designs for each gene target (Fig. 3a). Both gRNAs would target either the positive or the negative strand of the genomic DNA; each of the two gRNA would target either the 5’ or 3’end of the gene. The PAM-to-HA distance was mostly kept within 50 bp (Table 1). For the aHA design, the UP HA length was varied between 40–45 bp, whereas the DW HA length was kept at 500 bp. Out of the 27 gene targets, only *rodZ* was not successfully deleted, possibly because of an essential gene (*ispG*) being immediately downstream of *rodZ* [20]. The other 26 genes were successfully deleted by the CRASH protocol, and 18 out of 26 genes achieved ≥ 75% knockout efficiencies (Fig. 3c and Table 1). The data was clustered by target gene direction (forward or reverse), gRNA targeting strand (positive or negative) and proximate gene end (5’ or 3’), as shown in Fig. 3c. Unfortunately, there was no apparent pattern dictating the more efficient gRNA design. We then analysed the mean or median knockout efficiencies (Additional file 1: Table S3) and generalized the following heuristics for gRNA design: for the forward genes and the gRNAs targeting the negative strand, targeting 5’ end of the gene is preferred; for the forward genes and the gRNAs targeting the positive strand, targeting 3’ end of the gene is preferred; for the reverse genes and the gRNAs targeting the negative strand, targeting 3’ end of the gene is preferred; for the reverse genes and the gRNAs targeting the positive strand, targeting 5’ end of the gene is preferred (Fig. 3a and Additional file 1: Table S3). Notably, regardless of forward or reverse genes, the preferred gRNA targeting sites on the genome are similar: for the gRNA targeting the negative strand, proximate to UP HA is preferred; for the gRNA targeting the positive strand, proximate to DW HA is preferred (Fig. 3a). We have validated the heuristics with new gene targets and achieved successful deletions. Similar designs have been tested and successfully applied to other *E. coli* strains such as K12 MG1655 (results not shown). In addition, multiplex knockout in BL21 using CRASH was tested by deleting *adhE* and *ldhA* simultaneously. 87.5% colonies were successfully modified (Additional file 1: Fig. S1). It is noted that the creation of pTarget plasmids with multiple gRNA cassettes is time-consuming when repeating sequences (e.g. promoter and terminator regions) and possible secondary structures are involved. To apply the CRASH deletion protocol, we systematically deleted single and combinatorial genes in *E. coli* BL21 to push and pull more flux towards lycopene production.

### Increasing *E. coli* size

**Fig. 4:**
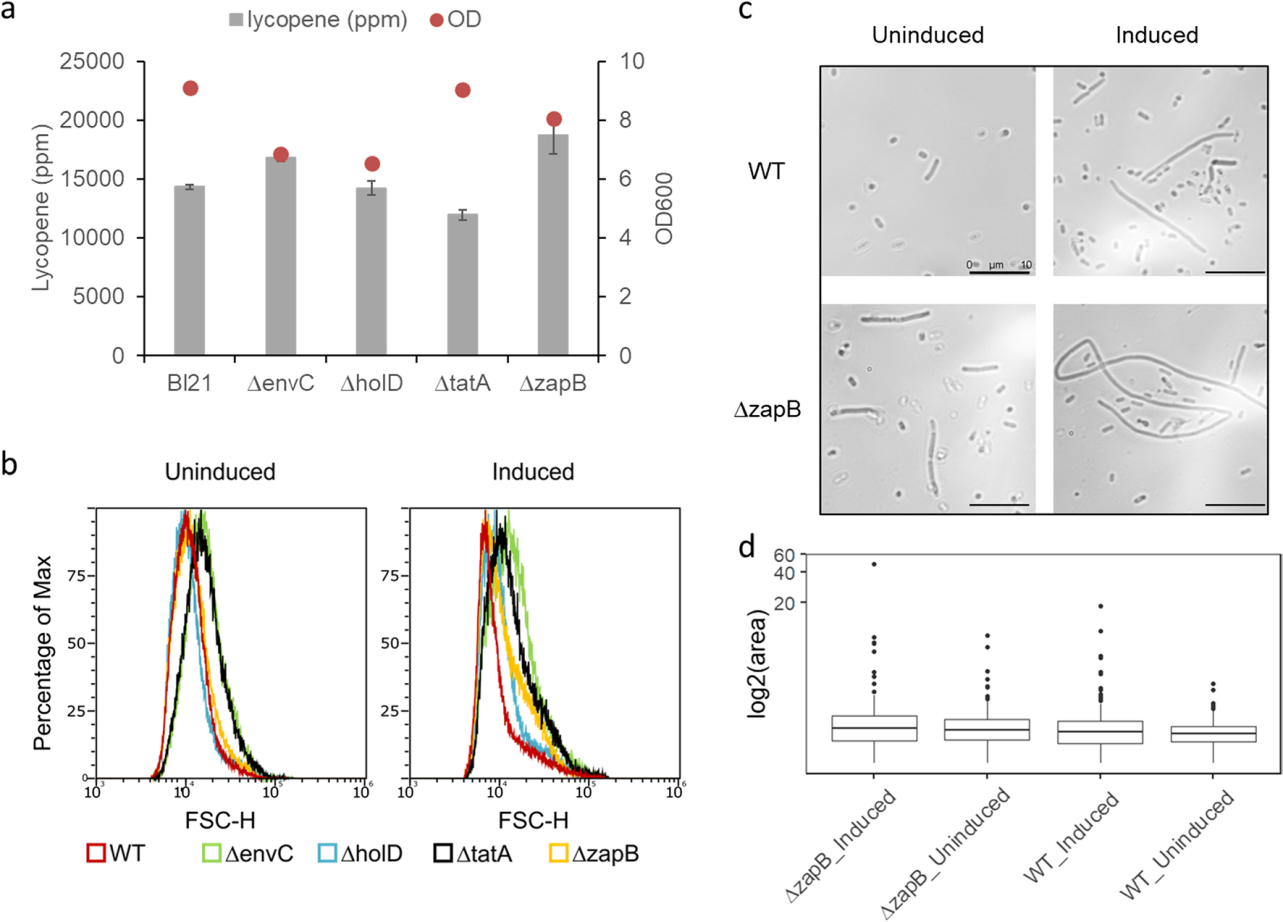
Effect of changing the cell size on lycopene production. **a** Specific lycopene yield and biomass of *E. coli* when genes affecting cell size were deleted*.* The lycopene pathway comprising module 1, 2 and 3 (Table 2) are overexpressed in each of single-deleted cells. All the measurements were average of triplicates with standard error bar shown in the figure. **b** Flow cytometry analysis on cell size distribution of single-deleted genotype. **c** Microscopy image of wildtype and *zapB* null strain with and without lycopene production. **d** Size distribution of wildtype and *zapB* null strain with and without lycopene production. They were obtained by imageJ analysis

**Fig. 5:**
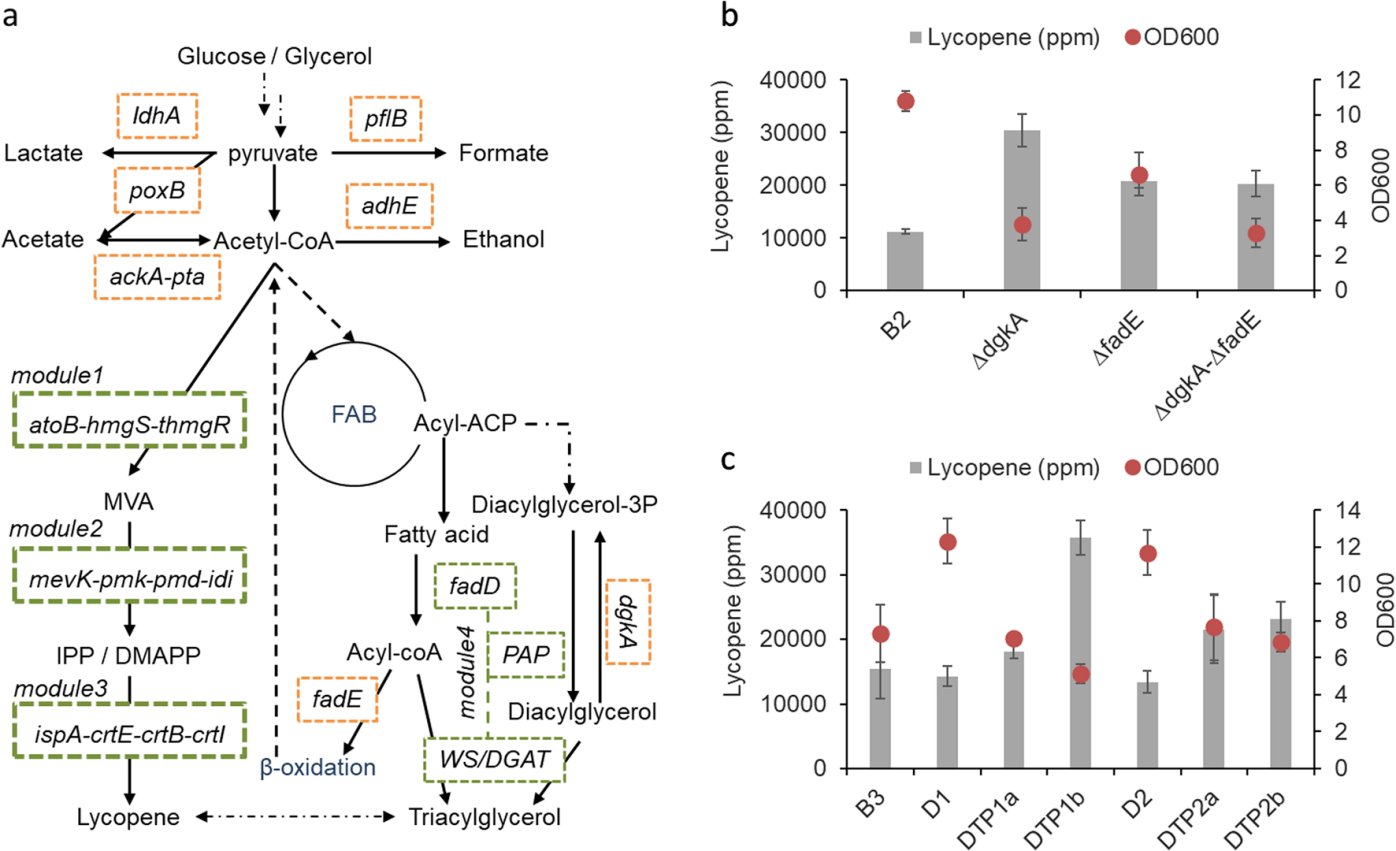
Engineering the triacylglycerol (TAG) pathway to increase lycopene production. a). Schematic representation of the TAG pathway and lycopene biosynthetic pathway. The two pathways are grouped into 4 modules, which is boxed with green dash lines (Table 2). The genes boxed in orange dash lines are targets to be deleted. The abbreviations are as follows. *adhE* alcohol dehydrogenase, *ldhA* lactate dehydrogenase, *poxB* pyruvate oxidase, *pflB* pyruvate-formate lyase, *pta* phosphate acetyltransferase, *ackA* acetate kinase, *atoB* Acetoacetyl-CoA thiolase, *hmgS* HMG-CoA synthase, *thmgR* truncated HMG-CoA reductase, *mevk* mevalonate kinase, *pmk* phosphomevalonate kinase, *pmd* mevalonate pyrophosphate decarboxylase, *idi* IPP isomerase, *ispA* FPP synthase, *crtE* GGPP synthase, *crtB* phytoene synthase, *crtI* phytoene desaturase, *FAB* fatty acid biosynthesis; *fadD* long-chain-fatty-acid—CoA ligase, *PAP* phosphatidic acid phosphatase, *WS/DGAT* wax ester synthase/diacylglycerol acyltransferase; *dgkA*, diacylglycerol kinase; *fadE*, acyl-coA dehydrogenase. **b** Specific lycopene yield and biomass of *E. coli* when *dgkA* and/or *fadE* were deleted. **c** Specific lycopene yield and biomass of *E. coli* when TAG pathway genes were overexpressed. All the measurements are averaged triplicates with a standard error bar shown in the figure

Lycopene was produced from an optimized mevalonate pathway as described in our previous studies [21–23] (Fig. 5a). Since lycopene is an intracellular compound, increasing cellular storage capacity may enhance the specific yield of lycopene [24, 25]. Thus, we hypothesized that altering the cell size might increase the storage capacity. To test this hypothesis, we selected four gene targets that have been shown to alter cell morphology upon deletion [26, 27]. EnvC activates the peptidoglycan (PG) amidases, regulates septal PG splitting and daughter cell separation; the *EnvC* null mutant results in improper cell division and forms long and chained cells [28, 29]. *HolD* encodes the DNA polymerase III subunit ψ. Deleting *HolD* affects DNA replication and cell division, leading to filamentous morphologies [26]. TatA is the part of the twin arginine translocation complex and transports the PG amidase to the periplasm, and the *TatA* deletion leads to the improper location of PG amidase, leading to long and chained bacteria [30]. ZapB is required for proper Z ring formation for cell division [27]. *ZapB* null strain displays an elongated cell shape. Using the CRASH protocol, we generated four single knockout strains and overexpressed the lycopene pathway genes in these cells. Lycopene content was measured after culturing the cells at 28 °C for 24 h. Interestingly, not all single knockout strains led to improved lycopene specific yield; only Δ*envC* and Δ*zapB* strains produced 17% and 30% more lycopene per cell, respectively (Fig. 4a). Moreover, the *envC* mutation led to much lower biomass, possibly attributed to the compromised cell envelope [29], whereas deleting *zapB* had little effect on biomass. To ascertain that these mutant strains indeed altered the cell morphologies, flow cytometry was used to gauge the size distribution for the wild-type and single knockout strains (Fig. 4b). When the lycopene pathway was not induced, the distributions for bacterial cells were generally gaussian. Both *envC* and *tatA* null strains displayed overlapping size distributions with significantly increased proportions of bigger cells. This is expected as both genes impacted the activity of PG amidase. The *zapB* null strain displayed similar size distribution as the wild-type except there was a slight increase in the percentage of bigger cells. The *holD* null strain unexpectedly displayed slightly reduced cell sizes, possibly because the cells were in a different growth phase due to a much slower growth rate. In contrast, when the lycopene pathway was induced, the size distributions became asymmetric, indicating heterogenous populations for all the strains tested. Interestingly, all the single knockout strains contained higher percentage of bigger cells as compared to the wild type. To verify this, we observed wild-type and Δ*zapB* strains under a phase contrast microscope and images were analysed with imageJ software. Without lycopene production, the wild-type strain remained rod-shaped, whereas Δ*zapB* strain was more heterogenous with long cells observed. In contrast, irregular and elongated cells were observed for lycopene-producing cells in both wild-type and Δ*zapB* strains, indicating that lycopene accumulation impacts cell division (Fig. 4c). Size distribution analysed by imageJ also showed that Δ*zapB* strain had an increased median size as compared to the wild-type strain (Fig. 4d). Increased heterogeneity or outliers were observed for lycopene-producing cells (Fig. 4d). To avoid plasmid instability issues due to heterogenous populations observed, we created an auxotrophic strain B2 by iteratively deleting semi-essential genes in addition to Δ*zapB* for subsequent lycopene production (Table 2) [31].

**Table 2:** Strains and plasmids used in this study

Name	Description	Reference	Remarks
*E. coli* BL21-Gold (DE3)	F^–^ *ompT hsdS* (*r*_*B*_^–^ *m*_*B*_^–^) *dcm*^+^ Tet^r^ *gal* λ(DE3) *endA* Hte	Stratagene	Base strain for genome editing
B2	*E. coli* BL21-Gold (DE3) Δ*aroA* Δ*aroB* Δ*aroC ΔserC ΔzapB*	This study	Auxotrophic strain with increased size
B3	B2 Δ*dgkA*	This study	Strain to increase TAG production
B4	B3 Δ*adhE*	This study	Strain to increase acetyl-coA
B5	B3 Δ*ldhA*	This study	Strain to increase acetyl-coA
B6	B3 Δ*adhE* Δ*ldhA*	This study	Strain to increase acetyl-coA
pTarget	Plasmid used to express sgRNA under J23119 promoter. *SacB* gene under its native promoter is inserted between aadA and pMB1 origin of replication	This study	Modified based on [6]
pCas-V5	Plasmid to express cas9 under its native promoter, λRed from pBAD promoter. It carries a temperature sensitive origin of replication and is kanamycin resistent. The sgRNA targeting pMB1 origin of replication is removed	This study	Modified based on [6]
pCas-V2	Similar to pCas-v5 except λRed is controlled by T7 promoter	This study	Modified based on [6]
pCas-V6	Similar to pCas-v5 with inactive *recA56* being overexpressed with *cas9* in a polycistronic manner	This study	Modified based on [6]
p15A-spec-Tm1-hmgS-atoB-hmgR	Plasmid for overexpression of *hmgs, atoB, thmgR* genes, controlled by mutated Tm1 promoter. It carries spectromycin resistance gene	[21]	Module 1
p15A-cam-Tm2-mevK-pmk-pmd-idi	Plasmid for overexpression of *mevK, pmK, pmd* and *idi* genes, controlled by Tm2 promoter. It carries chloramphenicol resistance gene	[21]	Module 2
p15A-kan-Tm1-crtEBI-ispA	Plasmid for overexpression of *crtE, crtB, crtI* and *ispA* genes, controlled by Tm1 promoter. It carries kanamycine resistance gene	[21]	Module 3
p15A-amp-Tm1-Ec.fadD (D1)	Plasmid for overexpression of *fadD* gene from *E. coli*, controlled by Tm1 promoter. It carries ampicilin resistance gene	This study	Module 4
p15A-amp-Tm1-Ec.fadD-a.DGT-Ro.PAP (DTP1a)	Plasmid for overexpression of *fadD* gene from *E. coli*, *WS/DGAT* from *Acinetobacter baylyi* and *PAP from Rhodococcus opacus,* controlled by Tm1 promoter. It carries ampicilin resistance gene	This study	Module 4
p15A-amp-Tm1-Ec.fadD-t.DGT-Ro.PAP (DTP1b)	Plasmid for overexpression of *fadD* gene from *E. coli*, *WS/DGAT* from *Thermomonospora curvata* and *PAP from Rhodococcus opacus,* controlled by Tm1 promoter. It carries ampicilin resistance gene	This study	Module 4
p15A-amp-Tm1-Ro.fadD (D2)	Plasmid for overexpression of *fadD* gene from *Rhodococcus opacus*, controlled by Tm1 promoter. It carries ampicilin resistance gene	This study	Module 4
p15A-amp-Tm1-Ro.fadD-a.DGT-Ro.PAP (DTP2a)	Plasmid for overexpression of *fadD* gene from Rhodococcus opacus, *WS/DGAT* from *Acinetobacter baylyi* and *PAP from Rhodococcus opacus,* controlled by Tm1 promoter. It carries ampicilin resistance gene	This study	Module 4
p15A-amp-Tm1-Ro.fadD-t.DGT-Ro.PAP (DTP2b)	Plasmid for overexpression of *fadD* gene from *Rhodococcus opacus, WS/DGAT* from *Thermomonospora curvata* and *PAP* from *Rhodococcus opacus*, controlled by Tm1 promoter. It carries ampicilin resistance gene	This study	Module 4

### Engineering triacylglycerol biosynthesis pathway

Another way to increase the storage capacity for lycopene is to engineer the neutral lipid pathway [25]. Previous reports have systematically optimized triacylglycerol (TAG) production in *E. coli*, which both *dgkA* and *fadE* genes were deleted, and TAG pathway genes were overexpressed (Fig. 5a) [32]. We tested single or double knockout of *dgkA* and *fadE* genes for lycopene production based on the B2 strain. As shown in Fig. 5b, all the three strains showed increased lycopene specific yield, especially *dgkA* null strain or B3 strain produced ~ 30,000 ppm lycopene. Even though the *fadE* null strain was beneficial to increase lycopene content, double knockout of *fadE* and *dgkA* did not yield a synergistic effect to improve the lycopene content further. Notably, we observed significant decrease in biomass when *dgkA* was deleted, possibly because of the accumulation of the toxic metabolite, diacylglycerol (Fig. 5b) [33]. Channelling diacylglycerol towards TAG may alleviate the toxicity. We thus overexpressed the TAG pathway genes as the 4th module in the B3 strain, which comprised long-chain-fatty-acid—CoA ligase (fadD), phosphatidic acid phosphatase (PAP) and wax ester synthase/diacylglycerol acyltransferase (WS/DGAT) (Fig. 5a). Two *fadD* and two *WS/DGAT* genes were screened [32]. As shown in Fig. 5c, when *fadD* from *E. coli*, *PAP* from *Rhodococcus opacus* and *WS/DGAT* from *Thermomonospora curvata* where overexpressed (DTP1b), lycopene content was further increased to ~ 35,000 ppm, even though biomass was not increased. It was noted that when only *fadD* was overexpressed (D1 and D2), B3 strain accumulated to higher biomass while lycopene content remained the same (Fig. 5c). This was possibly because flux towards diacylglycerol was partially diverted to fatty acid which could be re-converted back to acetyl-CoA via β-oxidation (Fig. 5a) [32]. This observation led us to hypothesize that acetyl-CoA availability may be limiting biomass accumulation [34].

### Increasing acetyl-CoA availability

**Fig. 6:**
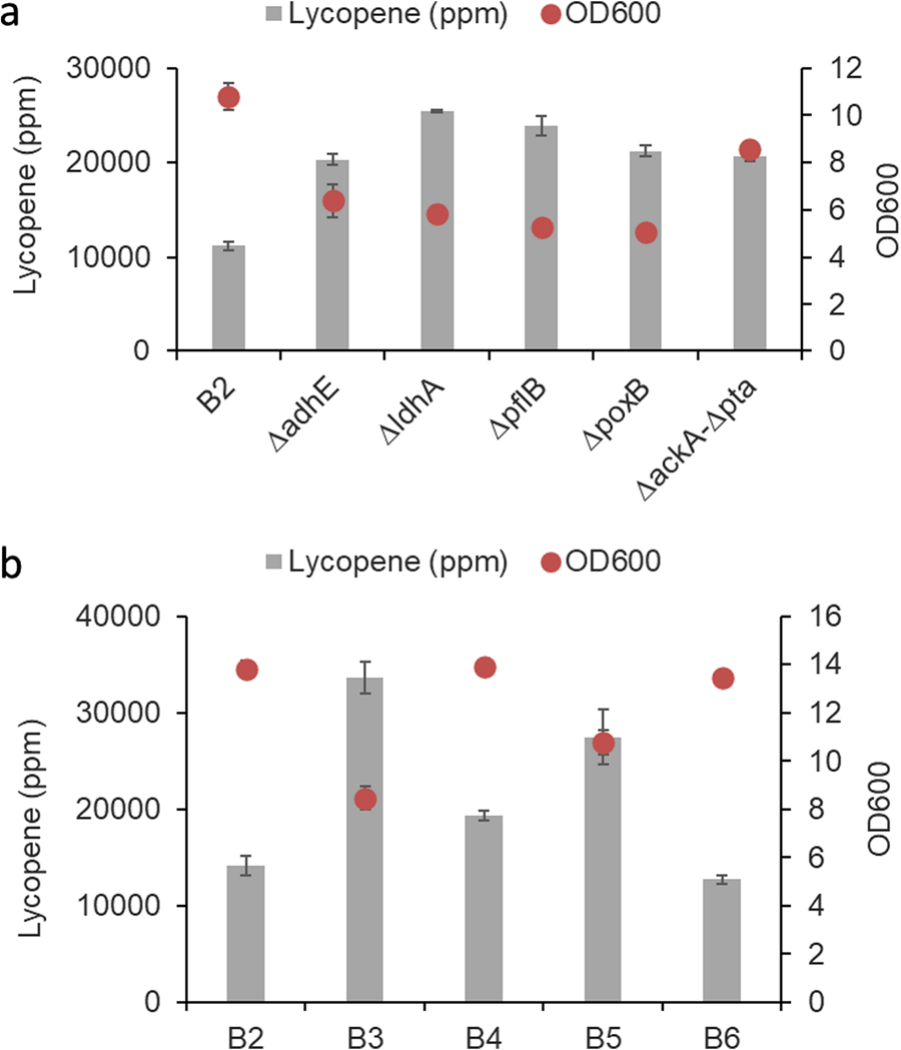
Increasing acetyl-CoA availability to improve lycopene production. **a** Specific lycopene yield and biomass of *E. coli* when divergent flux from pyruvate or acetyl-coA was deleted. **b** Specific lycopene yield and biomass of *E. coli* when combining both TAG overexpression and increasing acetyl-CoA availability. Refer to Table 2 for strain description. All the measurements are averaged triplicates with a standard error bar shown in the figure

To increase acetyl-CoA availability, we removed divergent flux from pyruvate and acetyl-CoA: namely *adhE*, alcohol dehydrogenase; *ldhA*, lactate dehydrogenase; *pflB*, pyruvate-formate lyase; *poxB*, pyruvate dehydrogenase; *ackA-pta*, acetate kinase and phosphate acetyltransferase (Fig. 5a) [36]. Single knockout strains based on B2 strain were created via CRASH. Subsequently, the three modules of lycopene pathway genes were overexpressed, and the strains were cultured in rich media for 3–4 days. As shown in Fig. 6a, all the single deleted strains produced higher specific yield of lycopene as compared to the B2 strain, partially attributed to the reduced biomass. Even though the *ackA-pta* deleted strain gave rise to the highest lycopene yield and biomass, the strain grew extremely slowly. Thus, we decided to test *adhE* and *ldhA* deletions which give the highest biomass and lycopene yield, respectively, among the remaining four single-deleted strains. Iterative gene knockout was carried out in the B3 strain to create single deleted—*adhE* (B4) or *ldhA* (B5)—strains and a double deleted—*adhE* and *ldhA* (B6)—strain (Table 2). All four modules were overexpressed with DTP1b as the 4th module. All the strains were cultured in rich media for 2 days. As expected, when *adhE* was deleted, acetyl-CoA could not be converted to ethanol, and the increased availability of acetyl-CoA improved the biomass of the B4 strain (Fig. 6b). Unfortunately, lycopene titer was not improved as compared to the B3 strain, leading to reduced lycopene specific yield in the B4 strain. Similarly, the *ldhA* null strain (B5 strain) increased pyruvate availability which activates pyruvate dehydrogenase allosterically to produce acetyl-CoA [35]. The B5 strain accumulated higher biomass and lycopene titer than the B3 strain, although lycopene specific yield was slightly decreased (Fig. 6b). Prolonging the culturing time to 4 days further increased lycopene titer of the B5 strain (~ 135 mg/L), resulting lycopene specific yield of ~ 42,000 ppm. Double deletion of *adhE* and *ldhA* did not show any synergistic effect on lycopene production, indicating additional factor(s) influencing lycopene production in addition to acetyl-CoA availability (Fig. 6b).

## Discussion

The field of metabolic engineering in *E. coli* has advanced significantly over the past decades. Impressive “TYR” (Titer, Yield and Rate) data have been achieved by modulating the metabolic pathway, improving the pathway enzymatic activities, and increasing co-factor availabilities [31, 34]. Recently, with the discovery and development of the CRISPR-Cas system, many studies have coupled the system with λRed recombineering to modify the host genome, redirect flux towards the desired products and minimize regulatory inhibitions [4, 37, 38]. In this study, we have further simplified the CRISPR-Cas9 mediated gene deletion protocol by using asymmetric homology arms as donor DNA. The donor DNA can be obtained by one single PCR step within 2 h. This avoids the tedious cloning steps involving multiple-piece gene assembly to carry the donor DNA on plasmid, or the use of overlapping PCR which requires optimization and gel purification (Additional file 1: Table S4) [16]. Moreover, our protocol only requires 100 ng of donor DNA, which can be obtained in sufficient quantity within 50 μl PCR reaction. More importantly, our protocol has achieved 100% knockout efficiency for deletion lengths of up to 3.4 kb, which is higher than previously reported (Additional file 1: Table S4). Each round of gene knockout took approximately 6 days, from cloning to plasmid curing (Additional file 1: Fig. S2). To further shorten the cloning time to half a day, we also tested directly transforming the PCR products of pTarget plasmid (with mutated gRNA region) and donor DNA into BL21 cells. One out of 8 randomly selected colonies was successfully deleted. The same method works with simultaneously deleting two genes on the genome, though the recombination efficiency was slightly decreased. It is worthwhile to note that generating the multiple gRNAs may become a bottleneck, and innovative solutions have been demonstrated such as CRISPathBrick [39]. Even though CRASH works for gene deletion, integrating DNA longer than 20 bp still requires cloning of the homology arms into the plasmid or overlapping PCR.

Another important aspect of CRISPR-Cas system is the gRNA design. Though in silico prediction tools provide a preliminary guide for the choice of gRNA, more fundamental studies are required to understand the efficiencies of gRNAs [12]. Here, we propose the general heuristics to design gRNA based on our CRASH protocol, where a slightly higher efficiency was observed when gRNA is targeting the positive strand of DNA nearer to the downstream homology arm or when gRNA is targeting the negative strand of DNA nearer to the upstream homology arm. Even though successful deletions can be achieved with heuristic gRNA design and CRASH protocol, knockout efficiencies vary for different genes and many “escaper” colonies have been observed. This warrants further mechanistic study to examine the non-edited cells which may further increase the knockout efficiency [12].

To apply the CRASH protocol, we tested 11 gene knockouts and iteratively knocked out 7 genes from the BL21 genome to improve lycopene production. Several recent studies have demonstrated that increasing the storage capacity of the microorganisms is beneficial to lycopene production [24, 25, 34]. Here, we demonstrated two more strategies to increase the storage capacity: firstly to increase the cell size and secondly to enhance the neutral lipid production. Heterogenous and elongated cell populations were observed when lycopene was accumulated in *E. coli*, suggesting lycopene may affect cell division. In fact, our initial size screening included Δ*rodZ* strain, which displayed reduced size of *E. coli*. We hypothesized that rodZ null strain may increase membrane surface to volume ratio, thus enhancing the storage capacity of the strain. However, we failed to knockout *rodZ* after a few attempts of changing gRNA and donor DNA designs. Though *rodZ* is not an essential gene, its downstream gene *ispG* is essential for cell growth. Deleting *rodZ* may affect ispG expression. Alternative approaches such as CRISPR interference or base mutation to repress or inactivate rodZ could be tested. In addition to regulating the cell size, our results show that overexpressing the neutral lipid pathway is helpful to enhance lycopene production, although it competes for the central metabolite acetyl-CoA [34]. Removing divergent flux from acetyl-CoA is shown to increase both the biomass and lycopene production. Pathway optimization between the lipid pathway and lycopene pathway have not been performed in this study, which potentially could be useful to further boost up the lycopene production. Taken together, with 6 rounds of screening, knocking out 16 genes and combining 7 deletions in the BL21 strain, we have improved the specific yield of lycopene by ~ threefold, from ~ 15,000 ppm to > 40,000 ppm. The strain requires further performance validation in the bioreactor.

## Methods

### Strain and plasmid construction

*E. coli* Bl21-Gold DE3 strain (Stratagene) was used in this study. The three lycopene plasmids were the same as previously described [21, 23]. The gene *fadD* from *E. coli* was amplified from the *E. coli* genome. The genes *fadD* from *Rhodococcus opacus*, *PAP* from *Rhodococcus opacus*, *WS/DGAT* from *Acinetobacter baylyi,* and *WS/DGAT* from *Thermomonospora curvata* were codon optimized and synthesized by Integrated DNA Technologies. They were cloned into the plasmid p15A-amp (L2-9) under mutant Tm1 promoter [21]. Plasmid and strain information is summarized in Table 2.

### CRISPR-Cas9 mediated gene deletion

Different pTarget plasmids with various sgRNAs were obtained by restriction free (RF) cloning methods [23]. The asymmetric homology arm (HA) donor DNA was amplified from the *E. coli* genome using iProof PCR mix (BioRad) and column purified by Zymoclean Gel DNA Recovery Kit (Zymo Research). Generally, 100–200 ng/μl of donor DNA in 30 μl can be obtained in a 100 μl PCR reaction. For the primer design, the forward primer is a fusion of the upstream homology arm (40–45 bp) sequence and downstream homology arm (15–20 bp) sequence (Fig. 1a and Additional file 1: Fig. S2). The 15–20 bp downstream homology arm is for annealing during initial cycles of PCR, and its length is chosen based on Tm ~ 50 °C. The total length of forward primer is kept at 60 bp. The reverse primer is a normal PCR primer about 15–20 bp with Tm ~ 50 °C. The length of downstream homology arms can be varied based on the reverse primer chosen. For this study, the downstream homology arm length was kept at 500 bp.

BL21 chemical competent cells were prepared using the Mix & Go! *E. coli* Transformation Kit (Zymo Research). For the construction of BL21 cells harbouring the pCas plasmid, 10 μl of cells were mixed with 50 ng/μl of pCas plasmid and heat shocked at 42 °C for 45 s. The cell was resucued in 200 μl of LB broth, at 30 °C, 300 rpm for 1 h before spreading onto LB agar containing kanamycin (50 μg/ml) and incubated overnight at 30 °C. A single colony was picked and inoculated into 1 ml LB medium containing kanamycin (50 μg/ml) and incubated at 30 °C, 300 rpm overnight for making electrocompetent cells.

For the preparation of electrocompetent cells, OD600 0.1 of the overnight BL21 cell culture harbouring the pCas plasmid was inoculated into 10 ml of LB medium containing kanamycin (50 μg/ml) and cultured at 30 °C, 300 rpm. 20 mM arabinose was added to the culture at OD600 0.2 for the induction of λ-Red recombinase. The bacterial cells were harvested at OD600 0.6 and centrifuged at 3800 rpm for 10 min at 4 °C. The supernatant was discarded and the cells were re-suspended in 10 ml 10% glycerol. The washing step was repeated twice. The electrocompetent cells was then suspended in 100 μl of 10% glycerol.

For electroporation, 20 μl of cells were mixed with 100 ng/μl of pTarget plasmid and 100 ng of donor DNA in the 1 mm Gene Pulser cuvette (Bio-Rad) and electroporated at 1.8 kV. The cells were rescued in 500 μl of LB broth, at 30 °C, 300 rpm for 3 h before spreading onto LB agar containing kanamycin (50 μg/ml) and spectinomycin (100 μg/ml) and incubated overnight at 30 °C. Colonies were screened by colony PCR using 2 × PCRBIO Ultra Mix (PCR Biosystems) along with an unedited BL21 strain as control.

The edited colony harbouring both the pTarget and pCas plasmids was inoculated into 1 ml of LB medium containing kanamycin (50 μg/ml) and spectinomycin (100 μg/ml) and incubated overnight at 30 °C, 300 rpm. For the curing of the pTarget plasmid, the culture was streaked onto LB agar containing 5% sucrose and kanamycin (50 μg/ml) and incubated overnight at 30 °C. The curing of the pTarget plasmid was confirmed by verifying the cell’s sensitivity to spectinomycin (100 μg/ml) before proceeding on to the next round of genome editing. For the curing of the pCas plasmid, the cells harbouring the pCas plasmid were streaked onto LB agar and incubated at 42 °C overnight. The curing of the pCas plasmid was confirmed by verifying the cell’s sensitivity to kanamycin (50 μg/ml). To cure both plasmids at the same time, the cells were plated on LB agar with 5% sucrose and incubated at 42 °C overnight.

### Media and culture conditions

All the cells were grown in modified autoinduction media (20 g/L Peptone, 10 g/L Yeast extract and 10 g/L NaCl), supplemented with 0.5 g/L glucose, 10 g/L glycerol, 30 mM lactose, 75 mM 4-(2-hydroxyethyl)-1-piperazineethanesulfonic acid (HEPES), and 0.5% Tween 80. Briefly, OD_600_ 0.1 cell from overnight culture was inoculated into 1 mL fresh media in 14 mL BD Falcon™ tube. Cells were incubated at 28 °C for 24 h or longer as indicated in the text before harvest. The media were supplemented with appropriate antibiotics (100 mg/L ampicillin, 34 mg/L chloramphenicol, 50 mg/L kanamycin and 100 mg/L spectinomycin) to maintain corresponding plasmids.

### Extraction and quantification of lycopene

Intracellular lycopene was extracted from cell pellets using HAE organic solvent, comprising hexane: acetone: ethanol ratio to be 2: 1: 1 by volume. Briefly, 10–50 µL bacterial culture was collected and centrifuged. The supernatant was discarded. 200 µL HAE buffer was then added to the cell pellets. The mixture was mixed and heated at 50 °C in a thermoshaker at 1000 rpm for 30 min, and further vortexed at room temperature for 30 min in order to completely extract the lycopene from the pellet. Subsequently, the mixture was centrifuged at 14,000 g for 10 min to pellet down the cell debris. 100 µL of the supernatant was added to 100 µL ethanol in a microplate reader and the absorbance at 472 nm was taken to calculate lycopene concentration against an external standard curve. The dry cell mass was correlated with OD_600_ with a coefficient of 0.42 gDCW/OD_600_ The specific yield of lycopene was calculated by dividing the lycopene concentration to the dry cell weight (Eq. 1).

1 $$lycopene\,specific\,yield \left(ppm\right)= \frac{lycopene\,titer\,({mgL}^{-1})}{OD600*0.42 ({gL}^{-1}) }\times 1000$$

### Microscope Image and Flow cytometry analysis

For the microscopy assay, 5 µL *E. coli* cells were mounted directly on microscope slides and observed immediately under the microscope. Microscopy was carried out by using a 100 × Leica HCX PL FLUOTAR oil objective lens on a Leica DM6000 B microscope. Images were acquired by Leica Application Suite X software. Image analysis was carried out by ImagJ software. Flow cytometry analysis was carried out with Attune™ NxT Flow Cytometer (ThermoFisher Scientific). Cell cultures were diluted 1000 × in deionised water and 100 μL cells were analysed at a speed of 12.5 µL/min. The scatter signal was recorded in logarithmic scale. Threshold values for forward scatter and side scatter were set at 1000 and 300, respectively to eliminate background signals from debris. The signal was gated using forward and side scatter to exclude non-singlet cells. The cytograms were drawn using Attune NxT Flow Cytometer software version 3 and edited using Inkscape.

## Supplementary Information

**Additional file 1.** Additional tables and figures.

## Data Availability

All data supporting the findings of this study are available in the article, Additional file, or upon request from the corresponding author.
