# Supplementary material for: Metabolic engineering of Escherichia coli BL21 strain using simplified CRISPR-Cas9 and asymmetric homology arms recombineering
Source: Microb Cell Fact. 2022 Feb 5;21:19. doi: 10.1186/s12934-022-01746-z (PMC8817497; doi:10.1186/s12934-022-01746-z)
Supplement: Supplementary file 1 — Additional file 1. Additional tables and figures. [file 12934_2022_1746_MOESM1_ESM.pdf]

Metabolic engineering of *Escherichia coli* BL21 strain using simplified CRISPR-Cas9 and Asymmetric Homology Arms recombineering

Sudha Shukal#, Xiao Hui Lim#, Congqiang Zhang, Xixian Chen*

*Singapore Institute of Food and Biotechnology Innovation (SIFBI), Agency for Science, Technology and Research (A*STAR), Singapore.*

# Both authors contribute equally.

* To whom correspondence should be addressed.

Chen Xixian: SIFBI, A*STAR, Proteos level 4, Singapore 138673;

Email: xixian_chen@sifbi.a-star.edu.sg or xixian.chen@outlook.sg

Additional file 1

Table S1. Knockout efficiencies of different gRNA designs to delete *adhE* from BL21 genome and its *in silico* prediction [15]. Synthego: https://www.synthego.com/

adhE	Knockout efficiencies	# Colonies	chopchop (based on K12)	on-target score (sythego)	Ranking (sythego)
gRNA1	1/15	729±158		0.417	94
gRNA2	10/16	3503±28	64.65	0.646	no rank
gRNA3	14/15	4666±6	37.71	0.377	no rank
gRNA4	12/16	923±112	57.03	0.57	79
gRNA5	0/16	944±211	53.35	0.534	78

Table S2. Effect of tuning the recombinase expression levels on knockout efficiencies. The gel images for the colony PCR results were shown. C: the PCR results using non-edited cells as template. Both the deleted and non-deleted (WT) sizes are indicated as arrows.   Refer to table 2 for the plasmid description.

adhE-gRNA	Cas Version	Inducers	Colony forming units	Knockout efficiencies
gRNA1	pCas-V5	20mM Arabinose	59	0%
	pCas-V6	20mM Arabinose	99	0%
	pCas-V2	0.1mM IPTG	0	-
	pCas-V2	No inducers	0	-
gRNA3	pCas-V5	20mM Arabinose	4320	100%
	pCas-V6	20mM Arabinose	5720	100%
	pCas-V2	0.1mM IPTG	180	100%
	pCas-V2	No inducers	162	100%

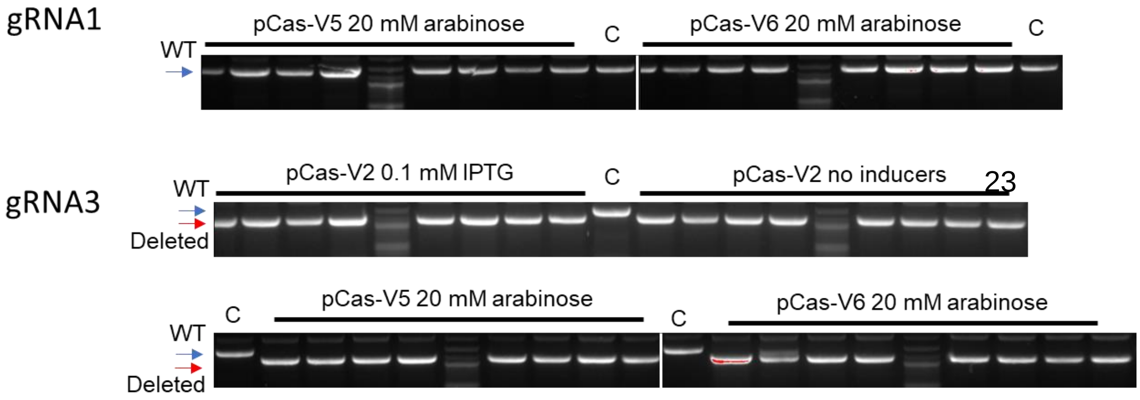

Table S3. Media and mean knockout efficiencies for the gene knockouts across the BL21 genome.

	Gene direction	gRNA targeting strand	Proximate gene end	Median KO efficiency	Mean KO efficiency
1	Forward	-	3'	0.625	0.62
2	Forward	-	5'	0.75	0.71
3	Forward	+	3'	0.5	0.46
4	Forward	+	5'	0.25	0.38
5	Reverse	-	3'	0.75	0.67
6	Reverse	-	5'	0.25	0.45
7	Reverse	+	3'	0.16	0.32
8	Reverse	+	5'	0.75	0.6

Table S4. Comparisons of genome editing methods tested in the *E. coli* BL21 strains by CRISPR-Cas system. pCas / pTargetF system, which our system is based on, is also included.

	pCas / pTargetF system	pEcCas / pEcgRNA system	pCasRed / pCRISPR-SacB-gRNA system	CRASH
Reference	Jiang et al. 2015	Li et al. 2021	Zerbini et al. 2017	Our work
Tested host	Mg1655<br>*Tatumella citrea*	BL21(DE3), BL21 Star^{TM} (DE3), MG1655, DH5α, CGMCC3705, Nissle1917, ATCC9637, DSM13699	BL21 ΔompA (DE3)	BL21 (DE3), MG1655
pCas or equivalent plasmid size	12545 bp	14605 bp	N.A.	11066 bp
Deletion length	N.A.	0.1-5.6 kb	0.03 - 2.3 kb	0.2-10 kb
Max length tested in BL21	N.A.	2.5 kb	2.3 kb	3.4 kb
Positive rate for the max length deleted	N.A.	1/2 (50%)	5/23 (14%)	2/2 (100%)

Duration (cloning, transformation and curing)	7.5 days	5.5 days	N.A.	6 days
Homology arm				
DNA fragment	Yes*	Yes	Yes	Yes
Amount transformed	400 ng	400 ng	1-10 µg	100 ng
Preparation time	≥ 4 h**	≥ 4 h**	> 1 day***	1.5-2 h
Generating the homology arm	overlapping PCR	overlapping PCR	Anneal 70-120nt synthetic DNA	one-step PCR
PCR products	Multiple bands****	Multiple bands	N.A.	Single band
Number of primers required	4	4	N.A.	2

*protocol mainly tested with homology arm cloned in pTargetF plasmid.

** Time includes PCR and purification

*** DNA synthesis and shipping takes 1 day

****DNA yield may suffer due to non-specific bands and gel purification

Figure S1. Multiplex knockout of *adhE* and *ldhA* genes simultaneously. The plasmid pTarget construct carrying 2 sgRNA expression cassettes is illustrated. Gel images of colony PCR results are shown. The same colonies were tested for both *adhE* and *ldhA* deletion. C: the PCR results using non-edited cells as template. Both the deleted and non-deleted (WT) size are indicated as arrows.

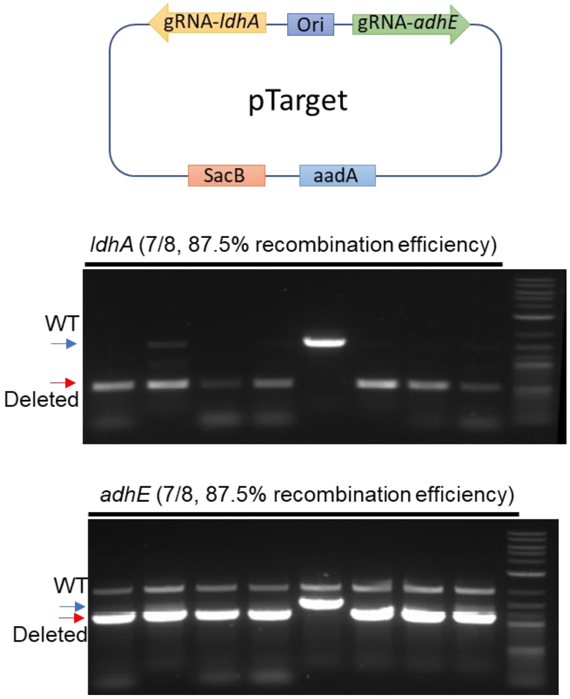

Figure S2. Illustration of the 6-day CRASH protocol. Please refer to materials and methods for details. The RF cloning method is used to mutate the gRNA sequences in the pTarget plasmid. To obtain asymmetric homology arms, the forward primer is a fusion of the upstream homology arm (40-45 bp) and downstream homology arm (15-20 bp). The 15-20 bp downstream homology arm is for annealing during initial cycles of PCR, and its length is chosen based on Tm ~50 ^{o}C. The reverse primer is a normal PCR primer about 15-20bp with Tm ~50°C. The length of downstream homology arms can be varied based on the reverse primer chosen. For this study, the downstream homology arm length was kept at 500 bp. While preparing the pTarget and homology arms, the pCas plasmid can be transformed into BL21 cells, and electrocompetent cells (ECC) can be prepared. Subsequently, the pTarget and homology arms can be transformed into BL21 cells overexpressing Cas9 protein and plate on LB + Spec + Kan agar. The next day, when colonies are formed, colony PCR is carried out to screen for positive clones. The positive clone is then inoculated into LB+Spec + Kan liquid media, and the cell culture is plated on LB+5% sucrose agar plate and incubated at 42 ^{o}C overnight to cure both plasmids. The cured colonies are then verified by testing their growth LB, LB+Spec, and LB+Kan (12h). Alternatively, the cell culture is plated on LB+Kan+5% sucrose agar plate and incubated at 42 ^{o}C overnight to cure the pTarget plasmid. The cured colonies can then be verified by testing their growth LB+Spec+Kan, and LB+Kan (12h).

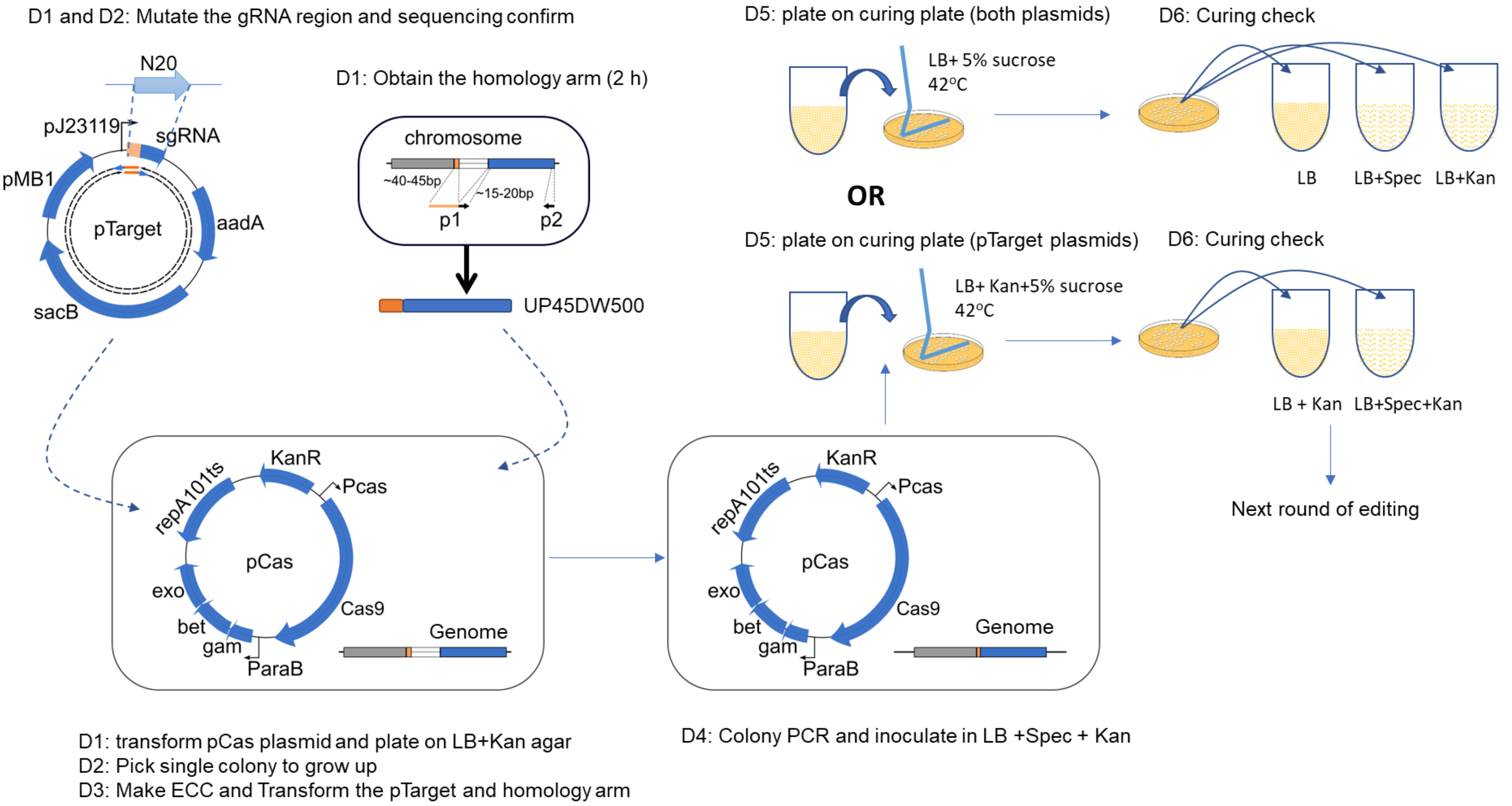
D1 and D2: Mutate the gRNA region and sequencing confirm
N20
pJ23119
sgRNA
pMB1
pTarget
aadA
sacB
D1: Obtain the homology arm (2 h)
chromosome
~40-45bp
~15-20bp
p1
p2
UP45DW500
D5: plate on curing plate (both plasmids)
LB+ 5% sucrose
42°C
D6: Curing check
LB
LB+Spec
LB+Kan
OR
D5: plate on curing plate (pTarget plasmids)
LB+ Kan+5% sucrose
42°C
D6: Curing check
LB + Kan
LB+Spec+Kan
Next round of editing
KanR
Pcas
repA101ts
pCas
exo
bet
gam
ParaB
Cas9
Genome
KanR
Pcas
repA101ts
pCas
exo
bet
gam
ParaB
Cas9
Genome
D1: transform pCas plasmid and plate on LB+Kan agar
D2: Pick single colony to grow up
D3: Make ECC and Transform the pTarget and homology arm
D4: Colony PCR and inoculate in LB +Spec + Kan
